# Panel Servitude: A Prophecy

**Published:** 1913-01-11

**Authors:** 


### PANEL SERVITUDE: A PROPHECY.

As a fighting speech on behalf of a body of men who feel themselves deeply wronged by the underhand and dishonest methods adopted for "stampeding" the doctors on to the panels, Mr. E. B. Turner's remarks at the Queen's Hall last Tuesday must have made even the modern politicians thankful that he is not in the House. The Chancellor's friends can gag the House of Commons by "blocking motions" and similar abuses of procedure; but they cannot gag Mr. Turner, whose refreshingly outspoken comments re-echoed in some passages the arguments, almost the exact language, of our leading article of a fortnight ago (The Hospital, December 28, 1912, p. 336).

At the moment of writing it seems as if the malign tactics of the Chancellor have met with a large measure of success, except in London, where it is clear that many of the panels will not be full. We do not envy him his success, such as it is, or the evil reputation which must inevitably attach to him. Every art of sophistry, deceit, intimidation, and corruption has been freely used to frighten or delude the doctors to join the panels. The Chancellor's satellites have not hesitated to advise that solemn pledges should be deliberately broken, and to assert that the pressure of "economic necessity" amply saves the personal honour of those who thus desert their written bond. To such disingenuity an unanswerable, if not very courteous, reply was made in one of the southern counties a few days ago by a greatly respected practitioner. At a meeting of all the best doctors in a country town, an emissary of Mr. Lloyd George offered to give five sufficient reasons why the pledge should be violated. To which the answer was, "Probably any lawyer could find fifty reasons for telling any lie or breaking any pledge; but we are gentlemen, and not lawyers."

The fact is, and it has got to be faced, that, with inconsiderable exceptions, the most efficient men in the profession are not going on the panels. The less efficient, less honourable, less trustworthy are joining them; and the public has noted that fact. Besides the stigma of inferiority which the panels now carry (owing to the methods of recruiting them), they signify also to the public broken faith and disloyalty. Upon this unquestionable fact those who have been forced or frightened on to the panels would do well to reflect. If in the future the profession finds itself divided into two classes, between which a great gulf is fixed, it will be due to the weakness and disunion of to-day. Apart altogether from the question of whether the British Medical Association was right or wrong, the doctors should have supported it loyally, from motives both of honour and self-interest. As it is the weaker brethren have merely split the Association, played into the hands of the wily demagogue George, who, in Mr. Turner's capital metaphor, has "passed like a malign comet, dragging behind him a daily lengthening tail of threats," and have earned for themselves the amused contempt of every section of the community. Events will show that those who have kept their word will stand higher in the public estimation than those who have broken it; and the latter will find themselves regarded pretty much on a level with the "parish doctor" of Dickens' day.

